# Barriers to healthcare and their relationship to well-being and social support for autistic adults during COVID-19

**DOI:** 10.1017/S1463423622000755

**Published:** 2022-12-14

**Authors:** Charlotte Featherstone, Richard A. Sharpe, Nick Axford, Sheena Asthana, Susan Ball, Kerryn Husk

**Affiliations:** 1 NIHR Applied Research Collaboration (ARC) South West Peninsula, University of Plymouth, Plymouth, UK; 2 Advanced Public Health Practitioner, Public Health, Cornwall Council TR1 3AY, UK; 3 European Centre for Environment and Human Health, University of Exeter Medical School, Knowledge Spa, Royal Cornwall Hospital, Truro, Cornwall TR1 3HD, UK; 4 Associate Professor, NIHR Applied Research Collaboration (ARC) South West Peninsula, University of Plymouth, Plymouth, UK; 5 Director of Plymouth Institute of Health and Care Research; Peninsula Medical School, University of Plymouth, Plymouth, UK; 6 Senior Research Fellow in Medical Statistics, NIHR Applied Research Collaboration (ARC) South West Peninsula, University of Exeter, Exeter, UK; 7 Senior Research Fellow, NIHR Applied Research Collaboration (ARC) South West Peninsula, University of Plymouth, Plymouth, UK

**Keywords:** autism, autistic adults, access to healthcare, health services delivery, COVID-19, emotional well-being

## Abstract

**Aim:**

The present study aimed to investigate barriers to healthcare and their relationships to social and emotional well-being and intersectional inequalities for autistic adults during COVID-19 restrictions in the UK.

**Background:**

Autistic adults experience severe health inequalities and report more barriers to accessing health services compared to other both disabled and non-disabled populations. The COVID-19 pandemic has impacted many areas of society that may have increased vulnerability of autistic people to social and health inequalities, including delivery of healthcare from in-person to remote methods.

**Method:**

One hundred twenty-eight autistic adults who lived in the UK took part in an online survey. Measures included the Barriers to Healthcare Checklist (Short Form) and PROMIS outcome measure bank to assess emotional well-being and social support. Participants rated their agreement with items, retrospectively considering three different points of the trajectory of COVID-19 restrictions: before COVID-19, during the first lockdown in spring 2020, and in the month prior to taking the survey during autumn 2020. They completed a follow-up survey six months later to continue to assess change as restrictions in the UK were eased.

**Findings:**

The average number of barriers to healthcare showed no significant change between all four time points. However, the nature of barriers to healthcare changed at the point of lockdown and persisted beyond the easing of COVID-19 restrictions. Barriers to healthcare were associated with some social and emotional well-being variables and demographic groups including gender, education and presence of additional disabilities. The findings may help to identify areas to target to improve access to both remote and in-person health systems for autistic people as modes of delivery continue to change over time.

## Background

The World Health Organization sets out that access to high standards of health and health resources is a human right (World Health Organization, 2017). Autistic adults have reported more barriers to accessing health services compared to non-autistic populations, including other disabled people without autism (Raymaker *et al.*, 2017). Barriers experienced by autistic adults include communication difficulties (such as problems using telephones), sensory issues in the healthcare environment, lack of provider knowledge about autism that may lead to misinterpretation and intra-personal factors such as executive dysfunction (Raymaker *et al.*, 2017; Mason *et al.*, 2019; Doherty *et al.*, 2022). The impacts of barriers to healthcare for autistic adults include delays to treatment and worsening of illness (Doherty *et al.*, 2022). This is concerning as autistic adults experience disparities in physical and mental health including higher prevalence of physical health conditions such as gastrointestinal problems, diabetes, epilepsy and cardiac illness, and mental health conditions, leading to significantly higher mortality rates than those seen in the general population (Croen *et al.*, 2015; Hirvikoski *et al.*, 2016; Woolfenden *et al.*, 2012). Healthcare access is important for identifying, monitoring and treating these conditions appropriately, as well as providing access to the pathway for autism diagnosis under the National Health Service (NHS) in the UK; accurate and timely autism diagnosis contributes to improved quality of life (Atherton *et al*., 2021).

Much of the previous research conceptualising barriers to healthcare for autistic adults was conducted prior to the COVID-19 pandemic. Restrictions on face-to-face contact, which began in the UK in late March 2020, introduced changes to health service delivery. This included services being delivered remotely and virtually (Webster, 2020). At the start of the pandemic, it was speculated that autistic people may be made more vulnerable to isolation, increased risk of mental health breakdown and reduced support from services (Cassidy *et al.*, 2020). Emerging research with this population has found that lockdown had a negative effect on mental well-being for autistic adults, including increasing anxiety and stress (Bal *et al.*, 2021; Pais and Knapp, 2021; Hedley *et al.*, 2021; Bundy *et al.*, 2022). However, qualitative studies revealed mixed impacts including positive experiences of a more stable routine, reduced social demands and increased access to healthcare and services due to remote and virtual delivery (Mosquera *et al.*, 2021; Hedley *et al.*, 2021; Bundy *et al.*, 2022). Challenges in healthcare included loss of contact and continuity from services, barriers to using remote healthcare and COVID-19-related services and reduced social support in health settings (Pais and Knapp, 2021; Bundy *et al.*, 2022). Mosquera *et al.* (2021) suggested that research should also aim to identify lessons learned from service delivery in lockdown that improved experiences for autistic adults.

### Aims and objectives of the present study

This study aimed to explore how barriers to accessing health services were affected by COVID-19 for autistic adults and their associations with emotional and social well-being. Based on prior research on barriers to healthcare, preliminary findings on the potential impact of the COVID-19 pandemic for autistic people (eg, Pavlopoulou *et al.*, 2020) and an expert roundtable discussion by Cassidy *et al.* (2020), we hypothesised (H1) that there would be a significant difference in the number of barriers to healthcare over time in accordance with the progression of the COVID-19 pandemic and (H2) that barriers to healthcare would be significantly associated with emotional and social well-being variables. Additionally, Cascio *et al.* (2020) have highlighted the need for autism research to attend to intersectionality, as some subgroups of the autistic population may have more specific support needs and associations with increased health disparities; for example, women and transgender people in this population have more pronounced disparities in morbidity and healthcare utilisation (Hall *et al.*, 2020; DaWalt *et al.*, 2021); Raymaker *et al.* (2017) explored the healthcare experiences of autistic people separately from those with disabilities other than autism, but did not assess the impact of co-occurring disabilities on access barriers for autistic people. Furthermore autistic adults experience disparities in education and employment (Brugha *et al.*, 2011; Office for National Statistics, 2022), but the impact of these on healthcare utilisation is underexplored. Cascio *et al.* (2020) argue that attending to intersectionality takes account of diversity in the autistic population, making research more applicable to the real world. We therefore aimed to include demographic factors in our analysis of the associations between barriers to healthcare and well-being to explore the influence of intersectional inequalities that may require additional attention in priority-setting for adapting health services.

## Methods

### Sample

Participants could take part if they were over 18 years old and had a formal autism diagnosis or if they suspected themselves to be on the autism spectrum. The research targeted a known hard-to-reach population as there is no record system of autistic people in the UK from which to draw a random sample. There is also a lack of definitive data on autism prevalence in adults as many autistic people do not receive a diagnosis until later life and some demographics such as women may be underrepresented in clinical diagnoses (Lai and Baron-Cohen, 2015). There were no clear sample size recommendations for the measures used when applied to survey methodology. Furthermore, the COVID-19 restrictions limited access to clinical samples. For these reasons, we used a convenience sample for this research.

The sample was limited to the UK to ensure consistency across health services and timings of pandemic control measures such as lockdowns. Participants were recruited primarily by a call for participants from Autistica’s Discover Network. In addition, we used filters from the National Autistic Society’s autism services directory (https://www.autism.org.uk/directory) to contact regional charities and services that supported autistic adults living independently, such as peer support and advocacy groups. We also asked regional branches of Mind, the mental health charity, to share the survey with service users. We contacted local authority Autism Partnership Boards and universities with disability societies or disabled student representatives identifiable using a Google search. The survey was also hosted online by several organisations and on our social media (see supplementary files – Recruitment Strategy for further details). The recruitment strategy was targeted at people who could participate in a survey independently or with minimal assistance, as we did not have the resources to adapt the survey for people with higher support needs without compromising validity of the measures.

### Materials

The following well-established questionnaires and measures were used to operationalise the relevant constructs in the survey:

***Barriers to healthcare checklist (short form)*** (Raymaker *et al.*, 2017) – a 17-item checklist coproduced with ‘autistic individuals, family members, health and disability services professionals, and academic scientists’ (Raymaker *et al.*, 2017), assessing barriers to accessing primary healthcare. The checklist was reviewed by Mason *et al.* (2019) and concluded to have high face validity and practical real-world applications, strengthened by its use of co-design methods. For the present survey, modifications were made to the questionnaire to improve applicability to a UK setting (removing a question regarding the cost of health insurance and changing ‘doctor’s office’ to ‘doctor’s surgery’ for cultural clarity). The tense of questions was also changed so that the scale could relate to multiple time periods.

***Patient-reported outcome measurement information system (PROMIS) measures relating to emotional well-being, social support and changes to routine:*** PROMIS is a publicly available bank of patient-reported outcome measures, aiming to capture outcomes most important to patients across medical conditions and contexts (Ader, 2007). These measures are completed by the individual and have good consistency across different methods of administration (Wang *et al.*, 2017). Holmes *et al.* (2020) developed the PROMIS Autism Battery–Lifespan (PAB-L), a bank of PROMIS measures chosen to assess quality of life across the lifespan in autistic samples. They found high feasibility and acceptability of these measures in a sample of autistic adults aged 18–65 years. Our questionnaire included scales relating to anxiety, depression, sleep impairment, satisfaction with social roles (eg, changes to work and home routines), and emotional and instrumental social support measures, which were most relevant to the types of challenges that participants might have encountered during lockdown (Cassidy *et al.*, 2020). Social well-being variables (social support and satisfaction with social roles) were measured at all time points as these were more objective, but we anticipated that emotional well-being (anxiety, depression and sleep impairment) could not be reliably recalled for the previous year due to their subjective nature and are excluded from pre-pandemic sections. PROMIS measures used a standardised *t*-score where the mean = 50 and SD = 10, based on representative samples from clinical and general populations in the United States (Cella *et al.*, 2010). Higher scores indicate better outcomes on measures of social well-being and poorer outcomes on emotional well-being scales.

***Screening questions:*** Participants indicated whether they had lived in the UK permanently since March 2020. Participants who self-identified as being autistic without providing details of a formal diagnosis completed the AQ-10 Autism Quotient (Allison *et al.*, 2012) to indicate whether they met the cut-off point of ≥6 indicating eligibility for autism assessment.

***Demographic questions included:*** Gender, ethnicity, household income, level of education, employment status, social deprivation by postcode area (as measured by Indices of Multiple Deprivation) (Ministry of Housing, Communities & Local Government, 2019) and presence of other disabilities (categories from the Office for National Statistics COVID-19 impact survey on people with disabilities, (Office For National Statistics, 2020)); attention deficit hyperactivity disorder (ADHD) was also added as another category due to high co-occurrence with autism (Ghirardi *et al.*, 2018) and COVID-19 related disability due to the context of the survey. These categories are self-reported and may not be associated with formal diagnosis.

These questionnaires formed part of a longer survey on well-being self-management during the COVID-19 pandemic, within a mixed-methods research project.

### Procedures

All research procedures were approved by the University of Plymouth Faculty of Health ethics board on 27/08/2020. The research explored experiences across four time periods (measured across an initial and follow-up survey) to relate findings to stages of COVID-19 restrictions. The initial survey, which was live between August-December 2020, asked participants retrospectively about their experiences in 2019 or before (pre-pandemic), during the initial UK lockdown period between March-May 2020 (hence referred to as spring 2020 reflecting UK seasonality), and during the past month (at point of survey completion between late August-December 2020, hence referred to as autumn 2020). A follow-up survey carried out six months after the close of the initial survey, from June to July 2021, related again to experiences over the previous month.

The survey was voluntary and took 30–60 minutes to complete, with the option to pause and return later to reduce overwhelm for participants which may encourage retention. Participants were required to read the information sheet for the study and agree to consent statements in order to proceed. Due to the inclusion of questions about emotional well-being, a debrief form provided participants with information about organisations which provide support and advice around emotional well-being and autism. All participants who had provided contact details (84% of the original sample) were contacted six months later up to three times with a prompt to complete the follow-up survey. The follow-up survey repeated the questions on barriers to healthcare and emotional and social well-being.

Due to COVID-19 restrictions, there were no face-to-face options for completing the survey; however, participants were informed that they could request support via phone or video call if required or request a printable PDF version of the survey to aid completion of the online form. The survey was piloted with four volunteers from a local adult autism support group, which led to changes including clarity of wording and structure of questions.

### Analysis

The data were analysed using IBM SPSS (version 25). Data on continuous variables were screened against normality criteria for linear models. We ran a repeated measures ANOVA to test hypothesis 1 (change in barriers to healthcare over time), with Greenhouse-Geisser correction. Linear regression modelling was used to test if social and, where applicable, emotional well-being variables, and demographic factors (gender, additional disabilities, education and employment), were significantly associated with barriers to healthcare (hypothesis 2). These models only included the responses of participants who had indicated a healthcare requirement for the relevant time point. The analyses were applied to two time points representing pre- and post-pandemic experiences (pre-2019 and autumn 2020) which were also considered to have the highest internal validity for all variables due to sample size and recall accuracy, respectively (although follow-up also used a similar recall time frame to autumn 2020, the sample was too small for regression analysis). Demographic categories were recoded into binaries where possible (employed vs. unemployed; university vs. non-university educated; additional disabilities vs. no additional disabilities) to ensure large enough group sizes for analysis. The PROMIS measures were analysed as continuous variables, as scores represented the sum of multiple Likert scales.

Chi-square tests were used to conduct further analysis of demographic variables that showed a relationship to barriers to healthcare in regression models, by analysing associations between demographics and each barrier in the checklist to identify specific issues affecting each group.

## Results

### Initial survey

#### Participants

One hundred twenty-eight participants completed the initial survey. 89.1% of the sample reported having a clinical diagnosis of autism. All of those without a clinical diagnosis scored above the cut-off of 6 or above on the AQ-10. Of those with an autism diagnosis, 76.6% had received this after the age of 18. The sample was mostly female (50.8% compared to 35.2% male and 13.3% nonbinary), white British (81.3%) and educated to university level or equivalent (69.5%), with 56% reporting being in paid employment. 35% of the sample reported having a household income of less than £20,000 p/a (the national median in 2020 was £29,900 – ONS, 2020) and 46% received financial support from the government.

Self-assessed co-occurring disabilities in the predetermined ONS categories (see Methods) included mental health (50.8% of the sample), learning disability or specific learning difficulty (20.3%), stamina, breathing or fatigue (19.5%), dexterity (15.6%), ADHD (15.6%), mobility (12.5%), memory (7.8%), hearing (6.3%), visual (3.9%) and pain (2.3%). Additionally within the ‘other’ category, 4.7% of participants self-disclosed sensory processing disorder, 2.3% gastrointestinal issues, 0.8% epilepsy, 0.8% COVID-related disability and 6.3% other disabilities. 23% reported no co-occurring disabilities.

Forty-two participants (39% of those re-contacted) completed the follow-up questionnaire. The follow-up sample had closer to equal numbers of males and females (47.6% male, 42.9% female and 9.5% nonbinary) compared to the original sample and was also older on average, with a higher level of disability. Educational level and employment status were proportionally similar to the initial sample. Table 1 shows the differences in demographics between the initial and follow-up cohorts.

**Table 1.** Demographics of initial and follow-up samples

Variable	***n*** **(%) Initial survey**	***n*** **(%) Follow up**
Clinical autism diagnosis	114 (89.1)	36 (85.7)
Non-diagnosis AQ Score >cut-off	14 (100)	6 (100)
Age		
18–25	11 (8.6)	<5
26–35	44 (34.4)	11 (26.2)
36–45	25 (19.5)	9 (21.4)
46–55	24 (18.8)	6 (14.3)
56–65	19 (14.8)	11 (26.2)
66+	5 (3.9)	<5
Approx. age at diagnosis (where applicable & stated)		
<18	26 (23.4)	8 (19.0)
18–25	25 (22.5)	<5
26–35	21 (18.9)	9 (21.4)
36–45	21 (18.9)	9 (21.4)
46–55	14 (12.6)	5 (11.9)
>55	<5	<5
Not stated	3 (2.6)	6 (14.3)
Gender		
Male	45 (35.2)	20 (47.6)
Female	65 (50.8)	18 (42.9)
Nonbinary/other	17 (13.3)	<5
Not stated	1 (0.8)	0
Ethnicity		
White British	104 (81.3)	34 (81.0)
White Irish	<5	0
Other White background	10 (7.8)	<5
White & Asian	<5	0
Other mixed background	<5	<5
Bangladeshi	<5	0
Caribbean	<5	0
Any other Black background	<5	0
Other	<5	<5
Not stated	4 (3.1)	0
Education		
No formal education	<5	<5
GCSEs/NVQ Level 1–2	17 (13.3)	6 (14.3)
A Level/NVQ Level 3	19 (14.8)	6 (14.3)
Undergraduate/Diploma/Equiv.	53 (41.4)	16 (38.1)
Postgraduate Degree	36 (28.1)	13 (31.0)
Not stated	1 (0.8)	0
Employment		
FT employment	47 (36.7)	14 (33.3)
PT employment	25 (19.5)	8 (19.0)
Retired	8 (6.3)	<5
Student	5 (3.9)	<5
Volunteer	10 (7.8)	5 (11.9)
Not in employment	30 (23.4)	10 (23.8)
Not stated	3 (2.3)	0
Disability (ONS categories)		
None	30 (23.4)	7 (16.7)
Visual	5 (3.9)	<5
Hearing	8 (6.3)	<5
Mobility	16 (12.5)	7 (16.7)
Dexterity	20 (15.6)	<5
Learning disability/SpLD	26 (20.3)	6 (14.3)
Memory	10 (7.8)	5 (11.9)
Mental health	65 (50.8)	27 (64.3)
Stamina, breathing or fatigue	25 (19.5)	10 (23.8)
ADHD	20 (15.6)	8 (19.0)
Other categories submitted:		
COVID-related disability	<5	0
Sensory processing disorders	6 (4.7)	0
Pain-related disability	<5	<5
Gastrointestinal disorders	<5	0
Epilepsy	<5	0
Others	8 (6.3)	<5
Receives government payments (eg, PIP, ESA)	59 (46.1)	20 (47.6)
Not stated	6 (4.7)	3 (7.1)
Supported by adult social care	18 (14.1)	9 (24.3)
Not stated	6 (4.7)	5 (11.9)

#### Healthcare use

Table 2 shows how participants in the sample required and used healthcare across the time points of interest. Those who indicated they had required health services were also asked to indicate if they had been offered remote healthcare during each time point. Approximately half of the sample had required healthcare at all time points. Use of remote healthcare increased from the pre-pandemic level of 23.4% of the sample, to over 40% since the onset of COVID-19.

**Table 2.** Healthcare use by sample

Time	***n*** **(%) of sample who required healthcare during time point**	***n*** **offered remote healthcare** (% of those who required healthcare)
2019 or before	128(100% assumed)	30 (23.4%)
Spring 2020 (lockdown)	71 (55.0%)	56 (78.8 %)
Autumn 2020	65 (50.8%)	45 (69.2%)
Summer 2021 (follow-up)	23 (54.8%)	17 (73.9%)

#### Hypothesis 1: barriers to healthcare would change over time

The data on measures used in statistical analyses showed some skew but were close enough to normality to use robust parametric tests.

Participants in the sample reported experiencing a mean of 9.83 barriers to healthcare prior to the COVID-19 lockdown (asked as ‘2019 or before’). Those who had accessed healthcare during lockdown and/or in the months prior to completing the initial and follow-up surveys reported 10.0 barriers during lockdown, 9.83 post-lockdown in autumn 2020, and 11.25 in summer 2021. These differences were not significant, *F*(1.88, 20.69) = 1.871, *p* = 0.181.

Table 3 shows the rankings of types of barriers experienced as percentages of the sample. The highest-ranking barriers at follow-up matched those during and post-lockdown 2020, although a greater proportion of the sample reported them than at previous time points (Table 2). Notably, Table 3 shows how the types of barriers experienced differed from pre-pandemic responses and persisted over time.

**Table 3.** Barriers to Healthcare Checklist (short form) categories with scores and ranks from present sample

Category	Items	% Scores				Rank			
		**2019 or before** ***n*** ** = 128**	**Spring 2020** ***n*** ** = 71**	**Autumn 2020** ***n*** ** = 65**	**Summer 2021** ***n*** ** = 23**	Time 1	Time 2	Time 3	Time 4
Emotional	Fear, anxiety, embarrassment or frustration kept me from getting to primary care	52.3	54.9	50.8	60.9	2=	4	4=	3=
Executive function	I had trouble following up on care	45.3	67.6	60.0	60.9	8	1	2	3=
	I had difficulty understanding how to translate medical information into concrete steps that I could take to improve my health.	28.1	35.2	36.9	47.8	14	13=	14	11=
Healthcare navigation	I felt that I don’t understand the healthcare system	37.5	45.1	49.2	56.5	11	8=	6	5=
	I found it too difficult to make appointments	48.4	63.4	61.5	78.3	6	2	1	1
	I had problems filling out paperwork	33.6	38.0	40.0	39.1	13	12	13	14=
Provider attitudes	My behaviours were misinterpreted by my provider or the staff	52.3	46.5	50.8	39.1	2=	7	4=	14=
	My providers or the staff did not take my communications seriously.	49.2	45.1	44.6	52.2	5	8=	10=	7=
	I could not find a healthcare provider who would accommodate my needs	43.0	53.5	43.1	43.5	9	5	12	13
	My providers or the staff did not include me in discussions about my health.	24.2	23.9	29.2	47.8	15	16	16	11=
Patient-provider communication	Communication with my healthcare provider or the staff was too difficult.	50.0	56.3	56.9	65.2	4	3	3	2
	When I experienced pain and/or other physical symptoms, I had difficulties identifying them and reporting them to my healthcare provider.	46.1	47.9	47.7	52.2	7	6	7=	7=
Sensory	Sensory discomforts (eg, the lights, smells, or sounds) got in the way of my healthcare.	39.8	35.2	44.6	52.2	10	13=	10=	7=
Socio-economic	I did not have a way to get to my doctor’s surgery	10.9	28.2	30.8	21.7	16	15	15	16
Support	I had inadequate social, family or caregiver support	35.2	45.1	46.2	52.2	12	8=	9	7=
Waiting	I found it hard to handle the waiting room	55.5	40.8	47.7	56.5	1	11	7=	5=
	**Total barriers (M)**	9.83	10.00	9.83	11.25				

#### Hypothesis 2: barriers to healthcare would be predicted by emotional well-being, social support and demographic variables

Table 4 shows the mean scores for the PROMIS variables against those reported in previous research in a similar sample by Holmes *et al.* (2020).

**Table 4.** Scores on PROMIS emotional and social well-being measures over time

Variable	**Mean, SD (** ***n*** ** = 128)**				**Average score in previous sample of autistic adults aged 18–65 (Graham Holmes *et al.* 2020)**
	2019	Spring 2020	Autumn 2020	Summer 2021	
**Emotional social support**	46.02, 11.55	45.47, 11.53	46.32, 10.36	44.13 (11.21)	48.1
**Instrumental social support**	46.47, 12.27	48.29, 13.65	47.21, 11.77	45.47 (10.86)	49.9
**Satisfaction with social roles**	46.27, 10.40	45.07, 11.01	43.91, 9.57	41.75 (9.09)	44.2^*^
**Anxiety**	N/a	64.41, 9.76	63.42, 10.27	65.30 (10.66)	60.9
**Depression**	N/a	60.98, 11.67	59.96, 11.45	61.49 (12.23)	57.8
**Sleep impairment**	N/a	56.88, 12.35	60.21, 10.74	60.72 (10.66)	64.4

* Alternative version of measure selected for present study (may not be directly comparable).

The regression model of pre-pandemic associations between demographics, social well-being and barriers to healthcare showed that gender, disability and satisfaction with social roles had significant associations with barriers to healthcare (Table 5), such that non-males, people with additional disabilities and those with lower satisfaction with social roles experienced more barriers. The regression model for autumn 2020 for associations between demographics, social and emotional well-being and barriers to healthcare demonstrated that education and anxiety had significant associations with barriers to healthcare (Table 6). People with a lower education level and higher anxiety experienced greater barriers. The associations between variables and the outcome in regression models were presented as estimated effects with 95% confidence intervals. Some multicollinearity was detected but VIF analysis suggested these were not of concern. Residuals were normally distributed in both models and casewise diagnostics suggested there were no serious outliers or undue influence of individual cases in either model.

**Table 5.** Associations with barriers to healthcare (2019 or before)

Variable	Estimated effect (95% CI)	***p*** **-value**
Gender	1.371 (0.32 to 2.43)	0.011^*^
Additional disabilities	−1.867 (−3.55 to −0.19)	0.030^*^
Education	−0.532 (−2.18 to 1.11)	0.523
Employment	0.009 (−1.55 to 1.56)	0.991
Instrumental support	0.013 (−0.06 to 0.09)	0.732
Emotional support	−0.065 (−0.14 to 0.14)	0.104
Satisfaction with social roles	−0.154 (−0.23 to 0.08)	<0.001^*^

* Significance (*p*) <0.05.

**Table 6.** Associations with barriers to healthcare (autumn 2020)

Variable	Estimated effect (95% CI)	***p*** **-value**
Gender	0.647 (-.86 to 2.15)	0.393
Additional disabilities	−1.304 (−4.05 to 1.44)	0.345
Education	−2.244 (−4.42 to −0.06)	0.044^*^
Employment	0.616 (−1.45 to 2.68)	0.552
Instrumental support	−0.028 (−0.13 to 0.08)	0.590
Emotional support	0.071 (-.05 to .20)	0.254
Satisfaction with social roles	−0.066 (−0.20 to 0.07)	0.320
Anxiety	0.204 (0.02 to 0.39)	0.032^*^
Depression	0.060 (−0.13 to 0.25)	0.518
Sleep impairment	0.080 (−0.03 to 0.19)	0.137

* Significance (*p*) <0.05.

Table 7 demonstrates analysis expanding upon the identified associations between demographic subgroups (gender, disability and education) and barriers to healthcare affecting this sample. At the pre-2019 time point, female and nonbinary participants experienced significantly more emotional difficulties (fear, anxiety, embarrassment or frustration), problems making appointments and following up on care, misinterpretation of behaviour by staff, feelings of not being taken seriously, difficulty identifying and reporting symptoms, inadequate social support and problems with waiting rooms, compared to males.

**Table 7.** Results from exploratory analysis of associations between Barriers to Healthcare Checklist totals and items with sample demographics

Barriers	**Pre-2019: Female/nonbinary (** ***n*** ** = 127)**	**Pre-2019: Additional disability indicated (** ***n*** ** = 128)**	Autumn 2020: Education status
Fear, anxiety, embarrassment or frustration kept me from getting to primary care	χ^2^(2) = 7.56, *p* = 0.023^*^	χ²(1) = 1.28, *p* = 0.259	χ²(1) = 0.65, *p* = 0.419
I had trouble following up on care	χ²(2) = 13.73, *p* = 0.001^*^	χ²(1) = 7.64, *p* = 0.006^*^	χ²(1) = 2.66, *p* = 0.103
I had difficulty understanding how to translate medical information into concrete steps that I could take to improve my health.	χ²(2) = 0.96, *p* = 0.650**	χ²(1) = 4.24, *p* = 0.039^*^	χ²(1) = 7.33, *p* = 0.007^*^
I felt that I don’t understand the healthcare system	χ²(2) = 2.38, *p* = 0.304	χ²(1) = 7.26, *p* = 0.007^*^	χ²(1) = 4.00, *p* = 0.046^*^
I found it too difficult to make appointments	χ²(2) = 6.12, *p* = 0.047^*^	χ²(1) = 0.05, *p* = 0.824	χ²(1) = 3.40, *p* = 0.066
I had problems filling out paperwork	χ²(2) = 4.56, *p* = 0.102	χ²(1) = 12.73, p<0.001^*^	χ²(1) = 18.50, p<0.001^*^
My behaviours were misinterpreted by my provider or the staff	χ²(2) = 16.08, *p* < 0.001^*^	χ²(1) = 2.39, *p* = 0.122	χ²(1) = 1.60, *p* = 0.206
My providers or the staff did not take my communications seriously.	χ²(2) = 10.14, *p* = 0.006^*^	χ²(1) = 0.54, *p* = 0.461	χ²(1) = 1.20, *p* = 0.273
I could not find a healthcare provider who would accommodate my needs	χ²(2) = 1.77, *p* = 0.414	χ²(1) = 11.06, *p* = 0.001^*^	χ²(1) = 1.56, *p* = 0.212
My providers or the staff did not include me in discussions about my health.	χ²(2) = 0.522, *p* = 0.808**	χ²(1) = 2.53, *p* = 0.112	χ²(1) = 0.51, *p* = 0.475
Communication with my healthcare provider or the staff was too difficult.	χ²(2) = 3.97, *p* = 0.138	χ²(1) = 8.53, *p* = 0.003^*^	χ²(1) = 5.68, *p* = 0.017^*^
When I experienced pain and/or other physical symptoms, I had difficulties identifying them and reporting them to my healthcare provider.	χ²(2) = 7.13, *p* = 0.028^*^	χ²(1) = 4.08, *p* = 0.043^*^	χ²(1) = 0.515, *p* = 0.473
Sensory discomforts (eg, the lights, smells, or sounds) got in the way of my healthcare.	χ²(2) = 5.28, *p* = 0.071	χ²(1) = 8.78, *p* = 0.003^*^	χ²(1) = 11.44, *p* = 0.001^*^
I did not have a way to get to my doctor’s surgery	χ²(2) = 0.90, *p* = 0.670**	χ²(1) = 4.81, *p* = 0.039^*^;**	χ²(1) = 1.15, *p* = 0.284
I had inadequate social, family or caregiver support	χ²(2) = 6.78, *p* = 0.034^*^	χ²(1) = 2.40, *p* = 0.121	χ²(1) = 1.01, *p* = 0.316
I found it hard to handle the waiting room	χ²(2) = 8.47, *p* = 0.014^*^	χ²(1) = 1.23, *p* = 0.268	χ²(1) = 2.87, *p* = 0.090

* Significant difference (*p* < 0.05);

** Fisher’s exact test used due to expected cell counts <5.

People who reported an additional disability experienced a mean score of 11 barriers to healthcare prior to the pandemic, while those without additional disabilities reported 3.5 barriers on average. Having at least one additional disability was significantly associated with reporting difficulty following up on care, translating healthcare recommendations into concrete steps, understanding the healthcare systems, filling out paperwork, accessing accommodations, communicating with providers, identifying and reporting symptoms, getting to a doctor’s surgery and sensory discomforts.

Analysis by education level in autumn 2020 showed significant associations between a lower level of education and understanding how to translate healthcare information into everyday steps to improve health, understanding the healthcare system, filling out paperwork, difficulties communicating and sensory problems.

## Discussion

This study used online survey methods to explore autistic adults’ experiences of accessing healthcare during COVID-19 and assess how healthcare barriers were associated both with emotional well-being and social support. The sample averages showed poorer emotional and social well-being compared to the standardised general population score (M = 50) on all well-being measures and across all time points, as indicated by lower scores on measures of social support and satisfaction with social roles and higher scores on measures of anxiety, depression and sleep impairment. They also showed poorer scores than those of Holmes *et al.*’s (2020) previous comparable sample of autistic adults, on all measures except for sleep impairment. The findings reflect previous research suggesting autistic adults experience low health-related quality of life (Holmes *et al.*, 2020; Oakley *et al.*, 2020).

The number of barriers to healthcare reported by this sample did not change significantly across the different stages of COVID-19 restrictions including pre-COVID, during lockdown and post-lockdown in 2020 and 2021. The results therefore did not support the hypothesis that the number of barriers to healthcare experienced would differ significantly across time. However, types of barriers reported during lockdown changed, and these changes persisted post-lockdown into the following year. These results may have implications for the future delivery of healthcare that retains methods of delivery such as remote consultations. The survey also found that after COVID-19, the number of barriers to healthcare experienced was significantly related to increased feelings associated with anxiety. Barriers to healthcare were also compared between differing demographic groups and some findings suggested there may be intersectional inequalities in accessing healthcare for autistic people.

Prior to lockdown, the highest-ranking barriers to healthcare access in this sample were waiting rooms, emotional concerns and misinterpretation by providers. Previous research supports the significant difficulty autistic adults experience in these areas compared to non-autistic populations (Raymaker *et al.*, 2017). In all time periods after lockdown, the highest-ranking barriers were difficulties following up on care, making appointments and communicating with providers. Previously, follow-up care was not a significant barrier for autistic adults compared to other groups (Raymaker *et al.*, 2017). This suggests lockdown restrictions may have made these aspects of healthcare more difficult for this population and that the easing of lockdown did not improve these experiences. This may be due to service closures, changes to guidance and the persistence of remote healthcare, as 58.6% of the sample who accessed healthcare reported receiving this in 2021 compared to 23.4% prior to the pandemic and 40.6% after the first lockdown in 2020. These findings suggest that more could be done by health services to improve methods of contact and communication with providers within the context of ongoing remote healthcare delivery and any continued restrictions on face-to-face contact, especially as autistic people experience existing disadvantage around communication with healthcare providers. It may also be worth exploring whether observed reductions in service use in the general population during the pandemic (Moynihan *et al.*, 2021) were due in any part to similar barriers around communication.

The direction of the relationship between barriers to healthcare and emotional and social well-being is not known due to the cross-sectional nature of the survey, but results suggested there was an association with satisfaction with social roles (eg, home and work routines) pre-pandemic and with anxiety post-pandemic. It may be that barriers to healthcare lead to decreased well-being or that poorer well-being may cause difficulties with access to healthcare, perhaps due to executive dysfunction, emotional regulation issues or communication problems.

Demographic factors including gender, additional disabilities and education were also found to have an association with barriers to healthcare. Prior to the pandemic, female and nonbinary participants were significantly more likely to report being misinterpreted by staff and not being taken seriously compared to males. This is supported by recent studies which also found that autistic adults identifying as women or ‘other’ gender experienced more barriers and unmet healthcare needs (Koffer Miller *et al.*, 2022). Autistic women are also at greater risk of many additional health conditions (DaWalt *et al.*, 2021) and suicide (Hirvikoski *et al.*, 2016), making this finding concerning. Higher level of disability was related to significantly more practical and communication-based problems compared to those with no additional disabilities. A survey by the Office For National Statistics (2020) found that during COVID-19, people with physical disabilities experienced more problems accessing medicine and other supplies. However, the associations between these variables and barriers to healthcare were not significant post-pandemic, which could indicate a levelling effect of more remote healthcare options for some marginalised groups. In autumn 2020 when there was a greater reliance on remote healthcare delivery, people with a lower level of education showed significantly more barriers around understanding healthcare information and navigating the health system; however, differences in group sizes for this variable mean caution may need to be applied to these findings. These findings suggest that people who experience increased marginalisation may be at a greater disadvantage in healthcare depending on external contexts.

### Strengths and limitations

This study has expanded on previous research by examining barriers to healthcare for autistic adults from an intersectional perspective, in the context of COVID-19 and in relation to emotional and social well-being outcomes. Many of the results were consistent with the findings of previous research. The measures for the main hypotheses had been previously validated in autistic adults (albeit in the United States, so required adaptation for the current sample and study purpose). The external validity and reliability of the study may be affected by the use of a self-selected convenience sample. The sample size achieved is associated with sampling error of between 7 and 10% (Williams, 2003); however, it is also likely the spread of demographics is not representative of autistic adults in general. For example, this group had a high level of female and nonbinary representation, although not at levels atypical for autism studies relying on anonymous self-report (eg, George and Stokes, 2018; Doherty *et al.*, 2022). Education and employment were also high compared to previous cohort studies aiming to identify a representative sample of autistic adults in the UK population (Brugha *et al.*, 2011). However, although nearly 70% of the sample were educated to university level, only 36.7% were currently in full-time employment, over 50% reported having a disabling mental health condition and almost half received financial support from the government. Therefore, although some subgroups of autistic adults in the UK population are less represented by this survey, the results highlight that even those who might be assumed to be more advantaged still experience concerning disadvantages with economic impacts, as appearing ‘high-functioning’ can itself represent a barrier for recognition of support needs in autistic adults (Wolfe, 2022). This is also the first study to our knowledge that has closely examined differences in barriers to healthcare across subgroups of gender, education and additional disabilities, showing where tailoring of services may be needed.

The survey sample was also not large enough overall to make inferences about the intersection of autism and some demographic groups such as ethnic minority status, especially as participants identified across a number of ethnic groups; condensing ethnic diversity into a binary provides a limited understanding of needs (Khunti *et al.*, 2020) and so was not applied to the analysis. Qualitative research and community participatory methods may be better placed to explore the experiences of minority groups’ access to healthcare to identify specific issues at this intersection of marginalisation and with specific minority groups in richer detail.

Although the survey aimed for consistency regarding the chronology of the pandemic by limiting responses to the UK, different decisions by devolved governments and the tiered system of restrictions introduced in Autumn 2020 by the UK central government, which changed often, may mean there are some inconsistencies across regions that could be present in the data and may have an unclear effect on results, affecting internal reliability.

### Recommendations for practice

The findings suggest that where remote delivery is in place, barriers may remain for autistic adults with communicating, understanding healthcare information and booking appointments, which may lead to delays in accessing healthcare and increase severity of health problems, potentially leading to more pressure on acute services. While Shaw *et al.* (2022) suggest remote delivery of services may have benefits for autistic patients, they also assert that adjustments are needed to overcome the barriers this poses, including offering online booking and a choice of methods for remote appointments to allow people to use their preferred method. In face-to-face care, improvements need to be made to the waiting environment. Previous researchers have suggested that enabling autistic patients to wait outside, and to reserve the first and last appointments, may ease anxiety around busy times of day (Mason *et al.*, 2021; Shaw *et al.*, 2022). The present survey also showed that healthcare interaction difficulties may have had an emotional toll for autistic people since COVID-19 which should be understood and adjusted for in consultations through patience, empathy and clear communication. Mason *et al.* (2021) found that an improved understanding of anxiety and compensatory strategies may help providers to be more accommodating of autistic patients. Providing information in advance of procedures may also help to reduce anxiety (Mason *et al.*, 2021).

Making changes to environments and communication methods will also benefit the wider population of healthcare users. For example, allowing multiple methods of contacting providers would also make using health systems more convenient and efficient for all patients. Furthermore, presentations of disabilities can overlap. As an example, making sensory adjustments and using clearer methods of communication could improve experiences for patients with conditions such as hearing and visual impairments and dementia. Additionally, undiagnosed autistic people would benefit from adjustments being made at a more universal level rather than implemented individually based on diagnosis. Involving autistic adults in local and central decision-making around accessibility in healthcare may also help to identify additional opportunities for improvement. Finally, the experiences reported disproportionately by women and nonbinary participants of the survey also reflect wider issues in the culture of healthcare around implicit bias in professionals’ adjudication of healthcare needs (Annandale *et al.*, 2007), which should continue to be identified and addressed.

### Conclusion

In conclusion, while remote healthcare has increased during the COVID-19 pandemic, the barriers to healthcare access for autistic adults have neither increased nor decreased but have shifted to enhanced issues around contacting and communicating with services. This is a concern for a population already disadvantaged by communication barriers. Barriers to healthcare were found to be related to some areas of emotional and social well-being for autistic adults across the lifespan and during COVID-19, and intersectional marginalisation may affect access to healthcare depending on context. This work may help to highlight areas that could require further attention in future research and practice to ensure equitable access to both remote and in-person health systems as modes of delivery continue to change over time.

## Supplementary material

For supplementary material accompanying this paper visit http://doi.org/10.1017/S1463423622000755. click here to view supplementary material
