# Supplementary material for: Barriers to healthcare and their relationship to well-being and social support for autistic adults during COVID-19
Source: Prim Health Care Res Dev. 2022 Dec 14;23:e79. doi: 10.1017/S1463423622000755 (PMC9817085; doi:10.1017/S1463423622000755)
Supplement: Supplementary file 1 [file S1463423622000755sup001.docx]

**Recruitment strategy**

| **Organisations** | **Actions** | **Total organisations contacted** | **Total organisations distributing survey** | **Reasons for not sharing (if given/applicable)** |
| --- | --- | --- | --- | --- |
| Autistica Discover Network | All members of network mailing list emailed | 1 | 1 | N/a |
| Autism support groups and charities | Organisations identified through National Autistic Society directory  Shared study invite and information with group members and clients  Some groups requested additional actions e.g. first author spoke to group over web call to explain more; authors sent blank PDF of survey to group in advance | 79 | 17 | - Did not respond - One organisation felt autism research did not lead to tangible benefit so did not share with members |
| Mind branches | All identifiable local branches on Mind directory contacted with request to share study invitation and information with service users. | 146 | 2 | - Did not respond - Against policy to share research requests - Did not have adequate resources |
| Autism partnership boards | Google search for “Autism” on sites ending “.gov.uk”  All identifiable boards contacted with request to share study invitation and information with board members | 22 | 7 | - Did not respond |
| University disability representatives | Google searches for “Disability” on sites ending “.ac.uk”  Disability support services, societies and student representatives on disability contacted with request to share study invitation and information to students and staff. | 22 | 3 | - Did not respond - Against policy to share research requests - Did not have adequate resources |
| SMARTEN (student mental health research website) | Shared study invite on public website | 1 | 1 | N/a |
| National Police Autism Association | Shared study invite on closed forum | 1 | 1 | N/a |
| Self-Care Forum (mental health website) | Shared study invite on public website | 1 | 1 | N/a |
| Healthwatch (regional patient involvement groups) | Shared study invite on closed intranet with instruction for regional groups to forward to relevant members | 152 regional groups (via intranet) | Unknown | N/a |
| Social media | 2 public posts on Twitter account | N/a | 29 retweets | N/a |
| National Autistic Society | Information sent to personal contact | 1 | 0 | - Already shared similar research |
| Special education colleges (natspec.org.uk) | Invite sent to central email address | 1 | 0 | - Did not have adequate resources and had many similar requests |
| Asian People’s Disability Alliance | Invite sent to contact email address | 1 | 0 | - Did not respond |
| Voiceability.org (advocacy service) | Invite sent to contact email address | 1 | 0 | - Did not respond |
